# Closed, Circular Genome Sequence of Aureococcus anophagefferens Virus, a Lytic Virus of a Brown Tide-Forming Alga

**DOI:** 10.1128/mra.00282-22

**Published:** 2022-06-09

**Authors:** Alexander R. Truchon, Eric R. Gann, Steven W. Wilhelm

**Affiliations:** a Department of Microbiology, University of Tennessee, Knoxville, Tennessee, USA; Queens College CUNY

## Abstract

Here, we report the genomic sequence of *Aureococcus anophagefferens* virus, assembled into one circular contig from both Nanopore and Illumina reads. The genome is 381,717 bp long with a GC content of 29.1%, which includes an additional 5-kb region between the previously predicted polar ends of the reference genome.

## ANNOUNCEMENT

We sequenced the genome of *Aureococcus anophagefferens* virus (AaV), a member of the family *Mimiviridae*, within the phylum *Nucleocytoviricota*. AaV has been propagated on its pelagophyte host, Aureococcus anophagefferens, since its isolation in the early 2000s and was first sequenced and assembled using Illumina reads in 2014 (1–3). Since then, the virus has been maintained via coculture with *A. anophagefferens* CCMP 1984. The original assembly was predicted to have terminal ends rich in leucine repeat-containing coding sequences (3, 4). As repetitive regions can lead to improper assemblies when exclusively employing short-read sequencing exclusively, we resequenced AaV using short- and long-read sequencing (5).

Viral DNA was extracted from a lysed, xenic *A. anophagefferens* culture as described by Truchon et al. (6). Briefly, virions were concentrated using tangential flow filtration and ultracentrifugation to enrich for virus particles. Particles were digested in agarose CHEF plug molds (Bio-Rad, Hercules, CA, USA) with proteinase K before being run on a low-melting point agarose gel. High-molecular-weight DNA was excised from the gel and purified using a phenol-chloroform method (6).

Long-read sequencing was performed using the Oxford Nanopore Technologies (ONT; Oxford, UK) platform. Genomic DNA libraries generated using the ligation sequencing kit (ONT) were sequenced on a MinION R9.4 flow cell (ONT), producing 284,000 reads that averaged 2,503 bp. Bases were called using the Guppy version 3.0.3 base caller using the config file dna_r9.4.1_450bps_fast.cfg (7). Adapter sequences were removed using Porechop version 0.2.4 (8), and the reads were trimmed with a quality score of 9 and a minimum length of 500 bp using NanoFilt version 2.7.1 (9). The reads were aligned to the AaV reference genome using BBMap version 38.90 (10) and used in the assembly, performed using Canu version 2.1 (11).

DNA was also extracted for short-read sequencing by treating concentrated virions with proteinase K for 1 h at 37°C and extraction via standard phenol-chloroform methods (12). The DNA library was prepared and sequenced on a NextSeq 2000 instrument (Illumina, San Diego, CA, USA) by the Microbial Genome Sequencing Center, generating 12,378,846 reads in 150-bp paired-end format. The Illumina short reads were trimmed for quality using the default settings in CLC Genomics Workbench (Qiagen, Hilden, Germany) and mapped to the Canu-assembled contig using Bowtie 2 version 2.2.3 (13). The assembly was polished with the Illumina reads using Pilon version 1.23 (14), which generated a closed, circular contig of 381,717 bp. The quality and completeness were assessed using CheckV (15). Coding sequences and tRNAs were predicted using Prodigal version 2.6.3 (16) and tRNAscan-SE version 2.0 (17), respectively.

**FIG 1:**
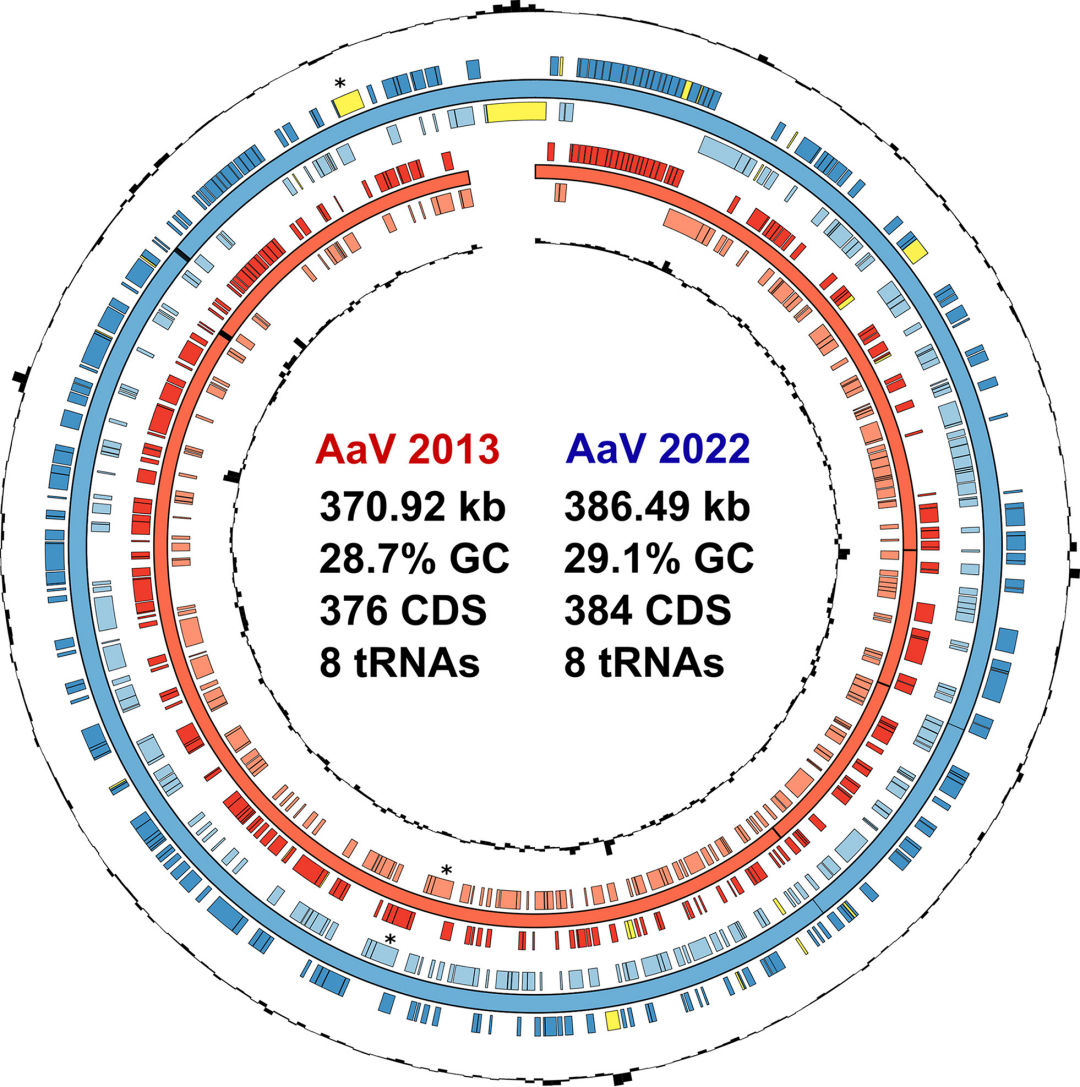
Genomic maps (created in Circos [20]) comparing the original AaV assembly (i.e., 2013) to the updated assembly (i.e., 2022). The complete genome sequences of the two assemblies were aligned, and the coding densities were compared. The rings (from inner to outer) indicate the GC content (2013), minus-strand coding sequences (2013), genomic sequence (2013), plus-strand coding sequences (2013), minus-strand coding sequences (2022), genomic sequence (2022), plus-strand coding sequences, and GC content (2022). Coding sequences present in only one genome are highlighted in yellow, and tRNAs are represented on the genomic sequence (2022) by black bands. Novel and known Rpb2 coding sequences are marked with an asterisk.

A total of 384 coding sequences (CDS) were predicted. Functions were predicted from translated amino acid sequences using the eggNOG-mapper Web server (18). One novel CDS encodes for a putative DNA-dependent RNA polymerase subunit (Rpb2). Only 1 of the 11 RNA polymerase genes encoded by AaV is homologous to the novel subunit, though the two sequences have an amino acid identity below 30%. This supports the hypothesis that two copies of this gene arose from an ancestral duplication among mimiviruses of eukaryotic phototrophs (19). Among other changes to the genome are apparent duplications, gene elongations, and gene combinations missed during the initial assembly (Fig. 1).

### Data availability.

The raw data and the assembled genome have been indexed at NCBI under the BioProject accession number PRJNA809211. The assembled genome has been assigned the GenBank accession number OM876856.1. The raw MinION and Illumina reads have been archived under the Sequence Read Archive accession numbers SRR16764708 to SRR16764709.
